# Development of drug resistance among HIV-1 F1 sub-type patients with treatment failure

**DOI:** 10.1186/1758-2652-13-S4-P142

**Published:** 2010-11-08

**Authors:** A Temereanca, CM Sultana, L Manolescu, C Vagu, C Grancea, S Ruta

**Affiliations:** 1 “Carol Davila” University of Medicine and Pharmacy, Bucharest, Romania; 2 “Stefan S. Nicolau” Virology Institute, Bucharest, Romania

## Background

The HIV/AIDS epidemic in Romania presents a series of particularities linked to the high frequency of a particular HIV subtype, a subtype that hasn't been found in other European countries, clade F, and by the existence of an important number of long life survivors patients infected during 1988-1992 - today young adults who presents a long history of treatment.

## Purpose of the study

In order to evaluate the development of drug resistant HIV strains, we sequenced the pol gene of HIV-1 isolates from heavily treated patients.

## Methods

The study included 26 HIV-1 isolates from heavily treated adolescents, with frequent changes in the antiretroviral combinations. Drug resistance genotyping was performed using the TruGene HIV-1 Genotyping Assay (Bayer Diagnostics). HIV subtype was determined using the Stanford database algorithm.

## Results

All the strains were found to belong to the F1 subtype. 65.4% of the patients presented resistance to at least one of the 3 antiretroviral classes of used drugs (NRTI, NNRTI and PIs ), while 15.4% were resistant to 2 classes and only 7,7% were resistant to all 3 antiretroviral classes. The most frequent substitutions of amino acids in reverse transcriptase gene were from TAM2's ("thymidine analogue mutations"): D67N, K70R, T215F, K219Q/E-mutations that confer resistance to NRTI and K103N, Y181C/I associated with primary resistance to NNRTI. Only 23% from the patients presented substitutions associated with major resistance to PI , the most frequent: I47V, G48 G/V; I54V, V82A, that confer phenotypic resistance to regimens that include ritonavir as a booster. As a surprise a series of minor and accessories mutations were present in the protease gene (ordered in decreased frequency: L10V, M36I; L63T, K20M/R).

## Conclusions

A follow up of the clinical progression rate of these patients will provide important data, as subtype specific resistance mutations have been reported and associated with different rate of CD4 cell count decline over time and with distinctive replicative fitness of the viral strain.

